# A District General Hospital Trauma Service Response to COVID-19: Lessons Learnt

**DOI:** 10.7759/cureus.12087

**Published:** 2020-12-14

**Authors:** Max Prokopenko, Harman Khatkar

**Affiliations:** 1 Plastic Surgery, Oxford University Hospitals NHS Foundation Trust, Oxford, GBR; 2 Orthopaedics, Royal Berkshire Hospital, Reading, GBR; 3 Nuffield Department of Orthopaedics, Rheumatology and Musculoskeletal Sciences, University of Oxford, Oxford, GBR

**Keywords:** general trauma surgery, covid 19, orthopaedics trauma, acute care surgery and trauma, covid-19 outbreak, return to elective service, covid-19 pandemic, orthopaedic disease

## Abstract

Objective: This article seeks to evaluate and outline the changes made to the trauma and orthopaedic department in a district general hospital in the United Kingdom during the COVID-19 pandemic. We detail the approach in relation to surgical management, workforce optimisation and our general reflections as a department.

Methods: We interviewed, collated and have subsequently described the adaptations implemented by our department. We have collected their shared strategy and reflections on how the COVID-19 pandemic affected our department.

Results: Alterations were implemented to mitigate the effects of the COVID-19 pandemic. A strategy focused on ensuring the workforce remained healthy, and patient care pathways were altered as minimally as possible.

Conclusions: As a unit, a sense of heightened vigilance needs to remain for the foreseeable future. Decisive action by departmental leadership, alongside a cohesive and open, has allowed for our trauma service to continue largely unchanged. This analysis serves as an important aide-memoire for future periods of extreme uncertainty.

## Introduction

The COVID-19 pandemic has altered the landscape of acute medical services provision worldwide. Healthcare systems globally have been forced to radically alter their standard operating procedures, at a local, regional and national level [1]. With the World Health Organisation announcement that the Coronavirus disease outbreak had become a pandemic on the 11th of March, hospitals across the United Kingdom took subsequent measures to prepare for the anticipated surge in cases, requiring the rapid implementation of service alteration in order to face the unprecedented challenge ahead [2]. Changes in service delivery have occurred across the primary, secondary and tertiary care settings. The purpose of this paper is to describe the changes implemented in response to the COVID-19 pandemic, serving as an aide-memoire for future periods of extreme uncertainty.

## Materials and methods

On consultation with the clinical lead for orthogeriatric and orthopaedic surgery, we collated and describe the changes employed by our department that facilitated a safe and effective continuation of Trauma service provision at a District General Hospital (DGH) serving a catchment area of 150,000. Additionally, we discuss the impact of these changes on the workforce, the resulting changes in rotas and staffing levels and the obstacles we encountered. We have retrospectively summarised our action within this article. With respect to quantitative operative data, online operative databases were interrogated to compared caseload between similar periods in 2019 and 2020.

## Results

Restructuring the team

In anticipation of the sick leave of medical colleagues, our department restructured existing rotas. Decisions made at a senior directorate level allowed our unit to acquire additional doctors of speciality training and specialist registrar grade from nearby tertiary sites to serve as a buffer for unpredictable absence due to COVID-19. These changes enabled the expansion of the workforce to staff theatre lists, fracture clinics, and the on-call service. We are fortunate to work alongside colleagues from separate tertiary elective units within the same NHS trust who can be re-deployed to staff acute services. Rota changes employed for doctors of all grades aimed to minimise staff footfall on-site in an attempt to reduce potential exposure to COVID-19. To reduce footfall and increase periods of rest, junior doctors were paired to work on-call rotas in teams of two, each covering a day and night shift respectively for four days in a row, followed by eight days rest. These periods of rest were felt to be protective of staff by allowing adequate recovery and enabled doctors to stay on standby, ready to step in and take over should any member of the on-call team become unwell and require time off work. 

The rapid mobilisation of the workforce to adapt to the growing COVID-19 demand has seen medical students graduate early, before completing hospital assistantship posts during medical school. Expediting the process of filling these positions allowed for a greater pool of doctors to the roster from [3]. After having a period of induction and phased introduction, including shadowing and supported practice, a Foundation Interim Year One (FiY1) doctor commenced work in the second week of May 2020. The doctor worked on their own specialised rota, primarily to learn and assist with basic ward duties. The advantage of this anticipatory start allows for familiarisation with the hospital environment, and the provision of further training. Practically, we hope this will ensure continuity and minimise disruption to services throughout the August rotation of doctors, a traditionally unsettling period for hospitals in the United Kingdom [4]. 

Adapting existing service pathways

At the beginning of the pandemic, we aimed to divert COVID-19 positive patients with traumatic musculoskeletal injuries to the acute service at a nearby tertiary centre, given that it has higher dependency care if required peri-operatively. Work done from the multicentre COVID-surgery study has clearly demonstrated that pre-operative COVID-19 infection is associated with pulmonary complications and significantly increased 30-day mortality, solidifying the decision to divert patients to tertiary sites [5]. 

**Figure 1:**
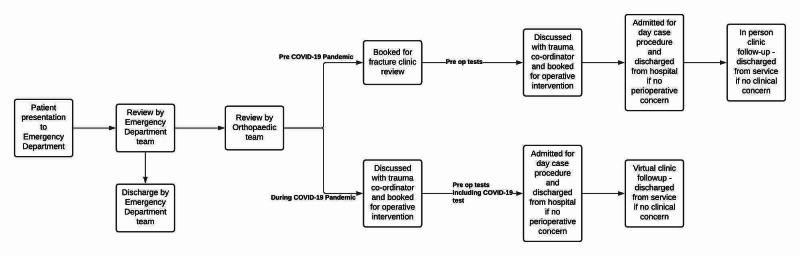
Ambulatory trauma patient pathway -- pre-COVID-19 pandemic vs during COVID-19 pandemic

A close working relationship between our team and the emergency department throughout the pandemic ensured patients presenting with trauma had prompt specialist input at the initial presentation. Initiating this change reduced the demand for fracture clinic appointments the subsequent day, decreasing footfall as patients were not required to return to the hospital for a second time. Specialist review in the emergency department at presentation was therefore equivalent to a new patient appointment. Further outpatient clinic review occurred at a later stage (Figure 1). 

Role of telemedicine

The use of telemedicine has come to the forefront of healthcare service provision during the pandemic. The introduction of social distancing rules and bans on mass gatherings was difficult for us to conduct trauma meetings with all members present due to the risk of COVID-19 transmission. Our unit utilised video conferencing to facilitate daily trauma meetings. All team members participated in the meeting from remote locations, limiting face-to-face contact in an attempt to reduce the risk of nosocomial transmission of COVID-19. The ability to virtually dial into the trauma meeting produced the added benefit of a larger cohort of senior doctors available to discuss the management of trauma cases, improving the expertise available. Challenges encountered include the quality of the video and sound, which relied on the existing hardware available in our unit. The technical barriers remain; however, we have learnt as a unit to adapt to this form of communication.

The trauma service also employed telephone calls for clinic follow-up appointments, by screening appointments appropriate for this facility. This facilitated remote review of patients without the added risk of transmission of COVID-19. Patients with a distinct clinical need for a face-to-face review are still invited to the clinic, however, they are counselled on the risks of attendance to the department. Personal protective equipment and strict adherence to disinfection and hygiene protocols were implemented for these clinics.

We did not institute a new patient virtual fracture clinic system. All virtual reviews were follow-up appointments for patients already reviewed by a specialist in person. All clinic telemedicine follow-up appointments were conducted by telephone calls, as we did not have the facilities to support video teleconferencing with patients during the pandemic. This is certainly an area to explore going forwards as we re-enter relative normality.

Changes to theatres and operative capacity

The beginning of the pandemic saw a requirement to restructure service provision at our DGH. Demand for expansion of medical services at our DGH required our trauma unit to relocate to an adjacent private hospital following NHS requisition of private facilities. We utilised existing infrastructure within this facility, repurposing planned elective surgery pathways to manage patients presenting with trauma. Structurally, this change resulted in the use of a single theatre commissioned for the sole purpose of managing trauma for the duration of the COVID-19 surge. Comparatively, prior to the pandemic, the DGH site operated four theatres, with trauma scheduled for an afternoon list in a single theatre each day. We utilised the private facility theatre scrub team for the duration of our transition as they were already skilled in managing elective orthopaedic cases. Relocation to the private facility doubled the operative time available each day with the availability of both a morning and afternoon list. However, time consumption ensuing from new protocols surrounding personal protective equipment (PPE) and aerosol-generating procedures (AGPs), meant that actual operative capacity reduced to approximately half. Elective services in our unit were postponed with immediate effect at the beginning of the pandemic. We continued to prioritise non-ambulatory trauma including patients admitted with femoral neck fractures. Ambulatory trauma service provision continued, with patients scheduled for theatre at the earliest available opportunity. 

**Figure 2:**
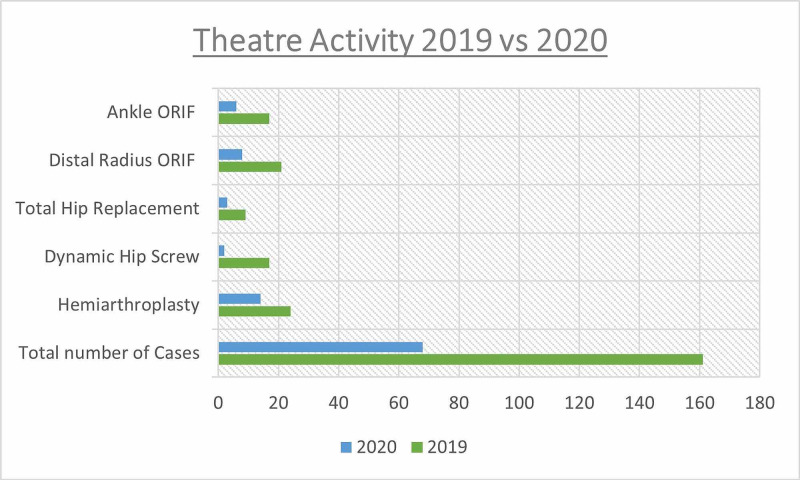
A graph demonstrating theatre activity from the third week in March through the first week in June -- 2019 vs 2020

Comparisons can be made between the present caseload to an equivalent period in 2019 (the third week of March through the first week of June). In comparison cases performed during the COVID pandemic were a third of the number performed a year previous (Figure 2).

 As a unit, we face similar challenges to all other surgical departments, knowing that elective cases need to be rescheduled in a fair and considered manner, whilst working through the sizable backlog.

Training opportunities 

Within our unit, theatre staffing was distilled down to essential personnel. Registrar attendance in the theatre was maintained throughout the COVID-19 pandemic, as the addition of a surgical assistant is critical for certain operations. The lack of elective operations within the DGH decreased the opportunity for junior colleagues interested in surgery to attend theatre and gain further knowledge of the operative environment. Medical training opportunities for both the orthogeriatric and surgical team were maintained, with regular attendance of the virtual daily trauma meeting encouraged for all members of the team. The unique COVID-19 environment allowed trainees to delve into other areas, such as developing leadership skills and partaking in audit and quality improvement. A focus has been on assessing how COVID-19 has affected our unit, and how we can learn and evolve in the future from this challenging period. 

Personal protective equipment 

Moving into the private facility, standard operating procedures were adopted from the private healthcare facility. Stringent adherence to Public Health England (PHE) and trust level PPE guidance was maintained. Staff working in the private facility prior to the COVID-19 pandemic followed a consistent approach to the use of PPE, ensuring that guidelines and protocols were followed accurately.

Junior doctors working in the unit received training in the correct use of PPE from the trust educational unit, eventually becoming trainers of correct PPE use. They worked as voluntary trainers, ensuring colleagues were cognisant with the fundamental techniques of donning and doffing. AGPs included the use of high-speed devices such as drills in theatre and endo-tracheal tube intubation and extubation. The protocol was to keep theatre doors closed continuously for twenty minutes after any AGPs inside the theatre were performed, allowing potential aerosols to settle prior to evacuating the theatre and thus preventing contamination of areas adjacent to the operating room. 

Changes to surgical management

Changes to operative management employed by our department aimed to minimise patient footfall within the hospital. We utilised absorbable sutures for closure of all operative wounds to prevent the necessity to return to the outpatient clinic in person. Additionally, for appropriate patients, we utilised plaster casts that could be removed by patients themselves at a later stage with clear instructions on how to do so. Doing so reduced the volume of patients presenting to the outpatient department. Further work is required to delineate whether these changes have affected the surgical outcome of patients.

## Discussion

Both junior and senior colleagues have been affected immeasurably by the current pandemic. The physical and psychological impact has been felt throughout our unit, and feelings felt have been reflected amongst the wider medical community [6].

Senior clinicians have had to consolidate the existing trauma service, implementing organisational structures and rotas with great speed. The temporary suspension of elective work has shifted the focus of the department to delivering urgent surgery and has thus allowed our unit to concentrate exclusively on delivering patient-centred trauma care.

Junior and senior colleagues described the initial period following the declaration of COVID-19 as a pandemic as alarming. Open discussions with senior colleagues and a culture of open communication allowed for frequent and useful troubleshooting. Throughout the pandemic, PPE provision has been deemed more than adequate for all team members at our unit, a source of great concern nationally for healthcare workers in the United Kingdom. Juniors have expressed disappointment in being unable to rotate to other specialties, however, some have also expressed satisfaction in being able to practice with continuity, an uncommon occurrence during the UK foundation program. This has allowed colleagues to fully realise the ‘firm structure’ in medicine, a concept that has become scarce in the UK healthcare system [7,8]. Continuing professional development for junior colleagues has been hampered during the crisis, with courses and exams postponed. 

A collective focus and strategy have promoted a team culture that we believe to be extremely positive and beneficial for our patients. The medical staff was required to work in unfamiliar environments, alongside allied health professionals with different professional experiences. The department has been able to absorb doctors previously working as resident medical officers for elective private patients, thus bolstering the medical support on the ward.

## Conclusions

The COVID-19 pandemic has truly illustrated that rapid mobilisation of existing services and decisive leadership strategy within the NHS is possible in times of great crisis. As a unit, we believe the lack of guidance at a national level at the beginning of the crisis hampered our ability to develop a rapid strategy to mitigate the effects of the pandemic. We need to remember the hard lessons learnt in this period. As a unit, we feel the time of crisis is far from over, and a sense of heightened vigilance and fortitude needs to remain for the foreseeable future. In the face of an unparalleled challenge, decisive action by departmental leadership, alongside a cohesive, open and honest strategy, has allowed for our trauma service to continue unchanged. 
